# Demographic Differences in Sun Protection Beliefs and Behavior: A Community-Based Study in Shanghai, China

**DOI:** 10.3390/ijerph120303232

**Published:** 2015-03-18

**Authors:** Shuxian Yan, Feng Xu, Chunxue Yang, Fei Li, Jing Fan, Linggao Wang, Minqiang Cai, Jianfeng Zhu, Haidong Kan, Jinhua Xu

**Affiliations:** 1 Department of Dermatology, Huashan Hospital, Fudan University, Shanghai 200040, China; E-Mails: shuxianyan0225@126.com (S.Y.); xufeng@medmail.com.cn (F.X.); feyfey252@hotmail.com (F.L.); lorrainejfan@gmail.com (J.F.); 2 School of Public Health, Key Lab of Public Health Safety of the Ministry of Education, Fudan University, Shanghai 200032, China; E-Mails: chunxue666@163.com (C.Y.); kanh@fudan.edu.cn (H.K.); 3 Department of Dermatology, Suzhou First Hospital, Anhui 234000, China; E-Mail: wlj234000@163.com; 4 Xinjing Community Health Service Center, Shanghai 200335, China; E-Mails: CmQ1008@163.com (M.C.); ag1887@sohu.com (J.Z.)

**Keywords:** knowledge, attitudes, behavior, sunburn, ultraviolet rays, skin type

## Abstract

*Objective*: We want to know the attitudes and behaviors towards UV protection and we want to analyze the difference between different Chinese demographic groups in this study. *Methods*: A community-based study was undertaken in Shanghai from October 2009 to January 2010. The participants, ages 20–60 years old, were screened by cluster sampling and were investigated through interviews at their own homes. Personal basic information and questions pertaining to their knowledge and attitudes towards sunlight and sun protective activities were included in the questionnaire. Results: We completed 5964 questionnaires (2794 men and 3170 women). Eighty-six percent of the respondents belonged to Fitzpatrick skin type IV. Knowledge about UV-induced risks was known by more than half of the participants. However, only one-third of the participants thought they needed sun protection in winter and indoors or in vehicles, and 27% of the participants acknowledged tanning was not favorable. The attitudes towards sun exposure varied greatly, showing significant differences based on gender, age, socioeconomic groups and skin type groups (*p* < 0.05). Fifty-five percent of the participants never use an umbrella under sunlight, only 26.5% of the respondents wear hats, and 21.3% of the participants applied sunscreen. Females and individuals of a younger age and higher education level were more likely to perform sun-protective behaviors than males and those of an older age and lower education level (*p* < 0.001). *Conclusion*: There is a deficit in the use of sun protection existing in our surveyed Chinese population, especially in males and lower socioeconomic population, which could allow for planning prevention campaigns and exploring sun-preventive products.

## 1. Introduction

There is increasing knowledge about the hazards of solar and ultraviolet radiation (UVR) to humans. It is well documented that ultraviolet (UV) radiation causes sunburn, premature aging of the skin, development of skin cancers and cataracts, immune suppression, and activation of latent viruses [1]. Some of the well-known skin conditions may be triggered or exacerbated by excess sun exposure, including actinic keratosis, basal cell skin cancer, squamous skin cancer, malignant melanoma, cutaneous lupus erythematosus, dermatomyositis, polymorphous light eruption, and disseminated actinic porokeratosis [2].

While climatological factors may influence the level of UVR at the earth’s surface, it is the behavior of people outside which has the greatest impact on personal exposure to UV. Consequent efforts are being made in Europe, U.S., and Australia to understand people’s attitude and behaviors towards the sun, allowing for the development of strategies to encourage limitation of their sun exposure to acceptable values [3,4]. These findings have promoted the development of some health education programs, and had a positive effect in reducing sunburn and the degree of skin photoaging, especially in reducing the rate of increase of the incidence of malignant non-melanoma skin cancer and melanoma in some areas [5]. However, there is little work being done in China, especially on skin cancer prevention, and most of the time, the harmful effects of UVR are ignored. Although the incidence of melanoma was much lower in China than that in the USA [6], actinic keratosis, basal cell carcinoma and squamous cell carcinoma are common skin cancers in China, which often affect those patients over 60 years old, and sometimes lead to a poor prognosis due to delay in diagnosis and treatment [7]. Furthermore, as Eastern culture is vastly different from Western culture, Chinese, especially women, always desire a light complexion, and tanning is not favorable. More and more Chinese are also increasingly concerned about the cosmetic problem, such as UV-induced skin aging [8], hyperpigmentation and seborrheic keratosis with the development of the economy. A community-based study showed that there was 100% prevalence of seborrheic keratosis in Chinese people over 60 years old [7]. To promote public understanding of UV protection and sun-smart projects under Chinese Eastern Culture, it is necessary to know the Chinese people's awareness, attitudes, and behaviors towards UV protection.

Only a few studies with small sample-sizes have been done in China. A simple random sample study of 680 people was conducted in Beijing, China in 2005 [9]. The results showed that 60% of participants had experienced sunburn and 40.6% of males expressed enjoyment while doing outdoor activities in strong sunlight. These results showed that people were lacking in general knowledge on UV damage. T-shirts and sun hats were the most common ways to avoid sunlight in males, who were less likely to use umbrellas and sunscreen, while females used a more diverse range of sun protection measures. A study including 623 volunteers from a North Chinese population in 2010 showed that although knowledge on the harmful effects of sun exposure was widespread, sun-protection measures were used limitedly. Sunscreens were employed by 58.8% of participants, followed by protective clothes (49.3%), sun umbrella (45.4%), sunglasses (45.3%), and hat (42.2%). Sex differences were observed explicitly [10]. A study among 385 medical undergraduate students in Shenyang, a city situated in Northeast China, found that even in a more knowledgeable group, UV was not actually comprehended thoroughly. Compared to men, women were more inclined to reduce sun exposure (*p* < 0.001) [11]. In recent years, with the rapid development of economic level and openness, the propaganda and advertisement of media and cosmetics companies have made people increasingly recognize hazardous effects of sunlight on the skin and the importance of sun protection in Shanghai, China. However, previous studies have shown that there is a deficit in sun protection knowledge, attitudes, and behaviors existing in Chinese population. It is clear that previous domestic studies were of a very small size, had relatively poor representation, and there is a lack of large-scale surveys in the Shanghai communities. 

Through a community-based study, we surveyed the knowledge and risk perception about solar radiation, attitudes towards sun exposure, behavior and duration of sun exposure, and use of recommended sun protective measures in the 5964 participants, ages 20–60 in Shanghai, east of China. Furthermore, we analyzed demographic differences (age, gender, education, income, and skin type) in sun exposure knowledge, attitude, and sun protection behaviors, and compared the differences that exist between the Chinese and Caucasians.

## 2. Patients and Method

### 2.1. Participants 

We conducted a cross sectional survey in the Xinjing Community, Changning District, Shanghai over a 4-month period from October 2009 through January 2010. The Xinjing Community is on the urban-rural boundary, and the distribution of age and gender in the population is similar to that of Shanghai. The residents, ages 20–60 years, living more than one year in the Xinjing Community, were screened by cluster sampling. Eleven residential blocks in Xinjing Community were selected. Questionnaires were completed during face-to-face interviews at their homes. A systematic check of the quality of the interviews was performed by calling back 6% of the participants. If this procedure revealed an abnormal finding in a single questionnaire, all the interviews conducted by the interviewer would be double-checked. No such abnormal finding was observed during the investigation.

### 2.2. Measures 

In addition to background variables, the questionnaires contained about 18 items, measuring knowledge about sun radiation, attitudes and risk perception towards sun exposure, sun protection behaviors, and frequency of sun exposure. The items used in the present paper were:

Background questions: the respondents were asked to indicate their gender, age, income, career, education level, and skin type. The education level was divided into three categories: Low (<high school graduate), Middle (high school graduate) and High (>junior college). Responses to the standard questions to determine Fitzpatrick skin types [12,13] were used as a self-report measure of sensitivity to sunburn and ease of tanning (see Table 1). Subjects were asked: which of the following best describes your reaction to exposure to late spring and early summer sun without sunscreen for about 45–60 min at midday? At the same time, the skin color of the inside part of the upper arm was checked. The responses to these questions were combined into six risk categories. 

**Table 1:** Fitzpatrick skin type method.

Skin Type	Susceptibility to Sunburn	Constitutive Skin Color	Facultative Tanning Ability
Ⅰ	High	White	Very poor
Ⅱ	High	White	Poor
Ⅲ	Moderate	White	Good
Ⅳ	Low	Olive	Very good
Ⅴ	Very low	Brown	Very good
Ⅵ	Very low	Black	Very good

Knowledge and risk perception about solar radiation: three questions concerned knowledge about sun protection and solar radiation. These questions were started with “*do you think sun exposure will*…”. The response alternatives were “*yes*” or “*no*”.

Attitudes towards sun exposure: the respondents were asked to indicate whether they needed sun protection in winter, and in vehicles or indoors. Another question was “*do you think tanning*…”. The response alternatives were “*appears healthy*”, “*looks* *attractive*”, “*is not favorable*”, or “*I do not care about this problem at all*”.

Behavior and duration of sun exposure: three questions concerning frequency and severity of sunburns during the daytime were asked. “*Do you avoid outdoor activities in the strong sunlight and avoid extensive sun exposure (more than 2 h) in the sunny mid-day?*”. Another question concerned the average amount of time spent in the sun, from 7:00 A.M. to 5:00 P.M. and from 10:00 A.M. to 2:00 P.M. 

Use of recommended sun protective measures such as minimizing exposure to the sun through wearing sunglasses, wearing long pants and a long-sleeved shirt, wearing a wide-brimmed hat, using an umbrella, staying in the shade, and applying sunscreen, are behaviors recommended in primary preventive campaigns. The likelihood of the respondents performing sun protection behaviors was measured by a 4-point scale of “*always*”, “*often*”, “*sometimes*,” or “*never*”.

### 2.3. Statistical Analyses

Data was entered on a relational database (EpiData 3.1) and the Statistical Package for Social Sciences (SPSS Version 16.0) was used for analysis. Participants were given a unique identifier, which was used to reconcile the data and remove any duplicates. The data was also classified by gender (female and male), age (20–34; 35–49; 50–60), income (low: <$3700/year; middle: ($3700–5600)/year; high: >$5600/year), educational attainment (low: <high school graduate; middle: high school graduate; high: >junior college) and skin type for all participants. Educational attainment and income levels were used as a surrogate indicator of socioeconomic status in some studies. First, we reported the frequencies for each major study variable. Then, we examined demographic differences (education, age, gender, and income) for these variables using univariate Chi-square analyses. All statistically significant values were two-sided with α = 0.05.

### 2.4. Ethical Considerations

The study protocol was approved by the ethics committee of Huashan Hospital, Fudan University, Shanghai, China. Informed consent was obtained from all participants.

## 3. Results

### 3.1. Characteristics of Participants

Eleven residential areas were selected in the Xinjing Community, with 13576 people, including 8322 residents, ages 20 to 60 years old. Of the 8322 participants given the questionnaire, 5964 (71.5%) participants responded. The background characteristics of the responders were shown in Table 2. There was an equal sex split and the group was diverse in terms of age (mean = 43.2 years), income and education, with almost 76% of participants practicing indoor jobs. Based on the answers given by the participants regarding their constitutive skin color and their skin reaction to solar radiation, skin type IV was the most common (86.2%), followed by skin type III (12%), and skin type I or II, which was only 1.8%.

**Table 2:** Characteristics of 5964 study participants who responded to a questionnaire on sun protection.

Demographic Characteristics	*NO.*	Participants (%)
Sex		
Male	2794	46.8
Female	3170	53.2
Age		
20–34	1923	32.2
35–49	1530	25.7
50–60	2511	42.1
Education		
Low (<high school graduate)	1767	29.6
Middle (high school graduate)	2146	36.0
High (>junior college)	2001	33.6
N.R.	50	0.8
Income ($/year)		
Low (<3700)	2191	36.7
Middle (3700–5600)	2053	34.4
High (>5600)	1635	27.4
N.R.	85	1.4
Skin type		
I or II	108	1.8
III	715	12.0
IV	5141	86.2

*N* = 5964. All percentages are weighted. N.R.: no response.

### 3.2. Knowledge and Risk Perception about Solar Radiation 

Sixty-seven percent of the responders knew that UV radiation contributes to premature skin aging, more than half of the participants (53.4%) knew that sun exposure leads to skin immune suppression, and 55.2% of the participants knew that solar radiation contributes to skin cancer. Knowledge about UV-induced risks was better known by younger adults, females, and those of a higher socioeconomic status (*p* < 0.001) as well as those with skin type I or II (*p* < 0.05) (Table 3).

**Table 3:** Knowledge about the UV-induced risk of the 5964 responders, stratified by age, gender, education level and income level.

Demographic Characteristics	Premature Aging	Immune Suppression	Skin Cancer
Age			
20 to 34 *n* = 1923	1439 (74.8%)	1227 (63.8%)	1272 (66.1%)
35 to 49 *n* = 1530	1033 (67.5%)	818 (53.5%)	845 (55.2%)
50 to 60 *n* = 2511	1522 (60.6%)	1147 (45.7%)	1173 (46.7%)
Age, *p* value	<0.001	<0.001	<0.001
Gender			
Male *n* = 2794	1669 (59.7%)	1336 (47.8%)	1404 (50.3%)
Female *n* = 3170	2324 (73.3%)	1855 (58.5%)	1885 (59.5%)
Gender, *p* value	<0.001	<0.001	<0.001
Educational level			
Low *n* = 1767	1037 (58.7%)	775 (43.9%)	827 (46.8%)
Middle *n* = 2146	1417 (66.0%)	1130 (52.7%)	1114 (51.9%)
High *n* = 2001	1503 (75.1%)	1253 (62.6%)	1320 (66.0%)
Educational level, *p* value	<0.001	<0.001	<0.001
Income			
Low *n* = 2191	1352 (61.7%)	1065 (48.6%)	1126 (51.4%)
Middle *n* = 2053	1369 (66.7%)	1111 (54.1%)	1075 (52.4%)
High *n* = 1635	1208 (73.9%)	966 (59.1%)	1035 (63.3%)
Income level, *p* value	<0.001	<0.001	<0.001
Skin type			
I or II *n* = 108	88 (81.5%)	72 (66.7%)	75 (69.4%)
III *n* = 715	462 (64.6%)	387 (54.1%)	407 (56.9%)
IV *n* = 5141	3443 (67.0)	2732 (53.1%)	2807 (54.6%)
Skin, *p* value	<0.001	0.014	0.023

### 3.3. Attitudes towards Sun Exposure

Thirty-six percent of participants thought that they needed sun protection in winter, while 28% of participants needed sun protection indoors or in the vehicle. Concerning the attitude towards skin tanning, more than half of participants did not care at all, 27% of participants thought it was not favorable, while only 5% of participants thought it looked attractive and 11% of participants thought it appeared healthy. The attitudes towards sun exposure varied with statistically significant differences in gender, age, and socioeconomic groups (*p* < 0.05) (Table 4). More females of a younger age and higher socioeconomic status thought they needed sun protection in winter, indoors, or in vehicles, and did not like tanning. A higher percentage of participants with skin type I or II thought they needed sun protection in winter, indoors or in vehicle than that of those with other skin types. (*p* < 0.05).

**Table 4:** Sun protection attitudes of the 5961 responders, stratified by age, sex, education level and income.

Demographic Characteristics	Need Sun Protection in Winter	Need Sun Protection Indoors or in the Vehicle	Do You Think Tanning		
			Appears Healthy	Looks Attractive	Not Favorable
Age					
20 to 34 *n* = 1920	961 (50.1%)	757 (39.4%)	195 (10.2%)	111 (5.8%)	666 (34.7%)
35 to 49 *n* = 1530	539 (35.2%)	417 (27.3%)	162 (10.6%)	80 (5.2%)	385 (25.2%)
50 to 60 *n* = 2511	643 (25.6%)	473 (18.8%)	274 (10.9%)	104 (4.1%)	540 (21.5%)
Age, *p* value	<0.001	<0.001	<0.001		
Gender					
Male *n* = 2793	776 (27.8%)	612 (21.9%)	307 (11.0%)	158 (5.7%)	382 (13.7%)
Female *n* = 3168	1366 (43.1)	1034 (32.6%)	324 (10.2%)	137 (4.3%)	1207 (38.1%)
Gender, *p* value	<0.001	<0.001	<0.001		
Educational level					
Low *n* = 1766	472 (26.7%)	363 (20.6%)	183 (10.4%)	89 (5.0%)	405 (22.9%)
Middle *n* = 2146	702 (32.7%)	528 (24.6%)	245 (11.4%)	96 (4.5%)	542 (25.3%)
High *n* = 1999	955 (47.8%)	746 (37.3%)	200 (10.0%)	108 (5.4%)	639 (32.0%)
Educational level, *p* value	<0.001	<0.001	<0.001		
Income					
Low *n* = 2189	719 (32.8%)	510 (23.3%)	252 (11.5%)	100 (4.6%)	547 (25.0%)
Middle *n* = 2052	718 (35.0%)	608 (29.6%)	224 (10.9%)	92 (4.5%)	563 (27.4%)
High *n* = 1635	673 (41.2%)	500 (30.6%)	152 (9.3%)	96 (5.9%)	454 (27.8%)
Income level, *p* value	<0.001	<0.001	0.008		
Skin type					
I or II *n* = 108	53 (49.1%)	53 (49.1%)	5 (4.6%)	10 (9.3%)	49 (45.4%)
III *n* = 715	289 (40.4%)	216 (30.2%)	63 (8.8%)	36 (5.0%)	214 (29.9%)
IV *n* = 5138	1800 (35.0%)	1377 (26.8%)	563 (11.0%)	249 (4.8%)	1326 (25.8%)
Skin , *p* value	0.002	<0.001	<0.001		

### 3.4. Behavior and Duration of Sun Exposure 

Seventy-seven percent of the participants avoided outdoor activities in strong sunlight and 78.7% of the participants avoided extensive exposure (more than two hours) in mid-sunny day. Female and individuals of a younger age and higher education level were more often performing sun-protective behaviors than males and those of an older age and lower education level (Table 5). Males reported spending more time outdoors than females during both 7:00 A.M.–5:00 P.M. and 10:00 A.M.–2:00 P.M. (*p* < 0.001). Those of an older age and lower education level were more likely to have long sunlight exposure than those of a younger age and higher education level (*p* < 0.001). But sun protection behaviors and sunlight exposure were not associated with income level (*p* > 0.05). A higher percentage of participants with skin type I or II avoid outdoor activities and extensive exposure to sunlight than other skin types.

**Table 5:** Behaviors and duration of sun exposure of the 5900 responders, stratified by age, sex, education level and income.

Demographic Characteristics	Avoid Outdoor Activities in Strong Sunlight	Avoid Extensive Exposure in Sunny Midday	Average Daily Sun Exposure Time (min)	
			7:00 A.M.–5:00 P.M. ^*^ 10:00 A.M.–2:00 P.M. ^*^	
AGE				
20 to 34 *n* = 1899	1522 (80.1%)	1560 (82.1%)	82.6 ± 71.9	21.8 ± 33.2
35 to 49 *n* = 1512	1149 (76.0%)	1176 (77.8%)	99.2 ± 95.3	26.5 ± 38.6
50 to 60 *n* = 2489	1869 (75.1%)	1904 (76.5%)	104.4 ± 92.3	27.2 ± 40.3
Age, *p* value	<0.001	<0.001	<0.001	<0.001
Gender				
Male *n* = 2760	1964 (71.2%)	1994 (72.2%)	111.8 ±105.7	31.1 ± 45.1
Female *n* = 3140	2574 (82.0%)	2644 (84.2%)	82.2 ± 64.7	20.1 ± 28.9
Gender, *p* value	<0.001	<0.001	<0.001	<0.001
Educational level				
Low *n* = 1753	1307 (74.6%)	1340 (76.4%)	111.2 ± 102.8	30.1 ± 45.9
Middle *n* = 2120	1623 (76.6%)	1649 (77.8%)	99.5 ± 92.3	26.1 ± 38.5
High *n* = 1977	1568 (79.3%)	1609 (81.4%)	79.1 ± 61.5	20.1 ± 27.2
Educational level, *p* value	0.002	0.001	<0.001	<0.001
Income				
Low *n* = 2164	1640 (75.8%)	1692 (78.2%)	96.0 ± 91.3	25.1 ± 40.1
Middle *n* = 2030	1585 (78.1%)	1606 (79.1%)	96.8 ± 86.6	24.6 ± 34.8
High *n* = 1621	1248 (77.0%)	1272 (78.5%)	94.8 ± 84.4	26.1 ± 37.5
Income level, *p* value	0.211	0.709	0.796	0.476
Skin type				
I or II *n* = 108	83 (76.9%)	88 (81.5%)	86.5 ± 88.3	43.5 ± 106.8
III *n* = 715	515 (72.0%)	516 (72.2%)	96.2 ± 104.2	58.0 ± 165.1
IV *n* = 5077	3940 (77.6%)	4034 (79.5%)	104.8 ± 121.9	47.4 ± 152.7
Skin , *p* value	0.040	<0.001	0.065	0.210

^*^ (mean ± SD).

### 3.5. Use of Recommended Sun Protective Measures

More than half of the participants in the survey never wear sunglasses, hats, long pants, or long-sleeved shirts to avoid sunlight. Furthermore, 55% of the participants never use an umbrella under sunlight, especially males. In the survey, staying in the shade was the most common way to protect the skin, and wearing long pants and long-sleeved shirts was the least used way. Only 26.5% of the respondents wear hats to avoid sunlight and 56.8% of those choose the brim of the hat between 2.5 cm and 7.5 cm.

**Figure 1:**
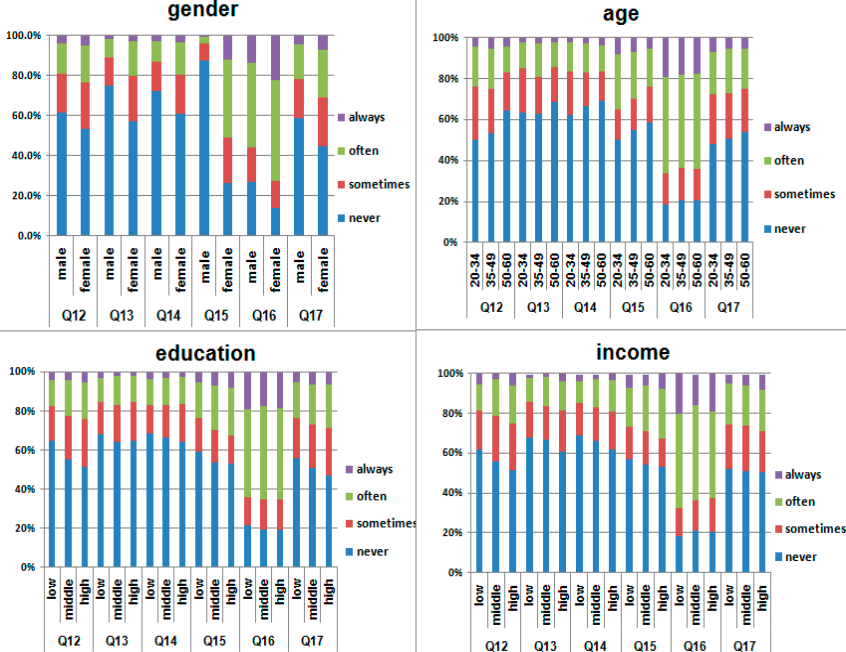
Different ways used by participants to protect them from sun, which were stratified by age, sex, education level and income. Q12: wear sunglasses; Q13: wear long pants and long-sleeved shirt to avoid sunburn; Q14: wear hats; Q15: use umbrella; Q16: seek shade while outside; Q17: consider the texture of sun-protective clothing.

The frequency of use of recommended sun protective measures varied significantly among gender, age and income, and education level groups (*p* < 0.05). More females, and those of a younger age or higher socioeconomic status, reportedly use several ways to protect the skin from the sunlight, while less male participants and those of an older age, lower education and income level, reported protecting themselves from sunshine (Figure 1). A higher percentage of participants with skin type III reported wearing sunglasses, hats, or long pants and long-sleeved shirts while exposed to sunlight, than those with skin type IV (*p* < 0.05).

In the survey, only 21.3% of the participants have applied sunscreen, 93.3% of which were female. Use of sunscreen was associated significantly with age, gender, socioeconomic status and skin type. Females and those of a younger age, higher socioeconomic level, and skin type III reportedly use sunscreen more frequently (*p* < 0.05).

## 4. Discussions

Sunlight is the main avoidable risk factor for skin disease. The Cancer Society and other organizations in U.S., Europe, and Australia recommend reducing exposures to UV, including using sunscreens, wearing protective clothing, staying in the shade, and avoiding exposure during the midday hours when the UVR from the sun is the strongest. So in our study, we tried to find out the practice of these behaviors in the Chinese from the community populations of Shanghai. The results showed that although more than half of the responders were aware of the UV-induced risk of skin cancer and premature aging, more than half of the participants in the survey never wear sunglasses, hats, or long pants and long-sleeved shirts to avoid sunlight. Fifty-five percent of the participants never use an umbrella, and nearly 80% of the participants never apply sunscreen under sunlight, especially the males. UVA radiation appears to play a key role in pigment changes occurring with age, and the major sign of skin photoaging in both winter and indoors in Asians [14], yet only one-third of the participants thought they needed sun protection in winter and indoors. Our results are consistent with the fact that improving the knowledge about the risk does not necessarily lead to optimal sun-protection behavior [15,16,17]. Although knowledge is essential to the practice of sun protection, it is by no means sufficient.

Factors such as gender, age, income and education attainment may represent a health status that conveys susceptibility, or they may be a predictor of socioeconomic status [18]. A study in U.S., including a general population national probability sample comprised of 1633 individuals with no skin cancer history, found that more accurate skin cancer beliefs and more adherent sun protection practices were reported by older individuals, and among those who were white and more highly educated. Women showed more active searching for health information and higher use of sunscreen and shade seeking; but men were more likely to use sun-protective clothing [19]. Another study among skin cancer-treated patients in France found there were no significant age-dependent differences in sun-protective behaviors [20]. Our study found that the differences in the knowledge, attitude, and behaviors of sun protection between different gender, age, income, and educational groups were significant (*p* < 0.05). Females and those of a younger age or higher socioeconomic status had more correct sun protection beliefs and were more willing to use several sun protection methods. The results of our study were a little bit different from that of the other studies. Maybe media campaigns and Internet use, which is now developing fast in China, promoted more younger people, females, people with a higher education level or higher income level, who were either more concerned with skin health or had better access to get the information, to care about skin protection from sunlight. On the other hand, our study found that the average daily sun exposure time of male participants during 7:00 A.M.–5:00 P.M. and 10:00 A.M.–2:00 P.M. was both significantly longer than that of female participants (*p* < 0.001). More than 70% of the male participants never wear hats or long pants and long-sleeved shirts to avoid sunlight, 88% of the male participants never use umbrellas, and 97% never use sunscreen; this suggests that there was a large need to improve sun protection and to help reduce photodamage signs among the male population in China.

The Chinese have different Fitzpatrick skin types from Caucasians. Eighty-six percent of our study population is Fitzpatrick skin type IV. At the same time, our study found significant differences in sun exposure attitude and sun protection behavior between skin type I and II and other skin types (*p* < 0.05). The present analysis confirmed other findings that those who reported to be more sensitive to sun exposure also reported that they were more likely to protect themselves from the sunlight [21]. So it was meaningful to make a well-organized, large-scale study to report the exact sun protection knowledge, attitude and behaviors of the Chinese.

As we anticipated, our study found that the attitude towards tanning of the Chinese with Fitzpatrick skin type III or IV was entirely different from that of the studies among Caucasians. Previous studies carried out within Europe and the U.S. have shown a similar trend that younger age groups, particularly young women, were also more likely to report feeling both healthier and more attractive with a sun tan and subsequently report sunburn and other skin cancer risk behaviors [22,23,24,25,26,27]. This also leads to a series of appearance-based interventions to reduce UV exposure and/or increase sun protection [28]. In our study, only about 15% of the participants considered tanning attractive or healthier, in contrast, 27% of the participants, especially young females, thought tanning was not favorable and avoided sun exposure in strong sunlight season, which could partially explain why younger females had the healthier knowledge, attitude, and behavior in our study. The different attitude towards tanning of the Chinese could also partially explain the fact that tanning beds, which are a growing public health concern in other parts of the world, has never been popular in China. As there are completely different standards of beauty, these results may also have some commercial value of exploring new beauty products for the Chinese market. 

Sun protection is increasingly being recognized as an important public health issue. But until now, there are no sun-smart schools or sun promotion projects or schools in China. Compared with the U.S. and European population [25], the surveyed population in our study has behavior defects, especially male and lower socioeconomic populations. With the development of the economy in recent years, more and more Chinese people are concerned about their appearance. In Asians, the principal manifestation of photodamage is pigmentary change rather than wrinkles; Seborrheic keratosis is the major pigmentary lesion in men [29]. So apart from the mass media, more future sun health promotion within the population is needed to improve knowledge and to encourage sun smart behaviors among men and those who are in lower socioeconomic status. 

## 5. Strengths and Limitations

The present study was the first to collect data on sun protection beliefs and behaviors from a large, community-based sample, with the sample age and sex distributions being similar to the Shanghai census distributions. The study was well organized and the questionnaires were completed by a trained investigator with the help of a dermatologist through face-to-face interviews. However, Self-reports were used in our study and recall bias might exit. Our results provided only an indirect assessment of sun protection practices among the population in Shanghai, east of China, as neither actual behaviors nor the amount of sun protection was measured. Although multiethnic populations exist in China, this study focused only on Han population. Given the size, diversity, and randomness of the sample, however, the findings have several implications for Chinese health.
